# Metabarcoding dataset on the elicitation of Soybean and Mungbean using *Ragi Tape* as elicitors for enhancing secondary metabolites production

**DOI:** 10.1016/j.dib.2022.108209

**Published:** 2022-04-26

**Authors:** Feri E. Hermanto, Warsito Warsito, Muhaimin Rifa'i, Nashi Widodo, Yoga D. Jatmiko

**Affiliations:** a Department of Biology, Faculty of Mathematics and Natural Sciences, Brawijaya University. Jl. Veteran Malang 65145, East Java, Indonesia; b Department of Chemistry, Faculty of Mathematics and Natural Sciences, Brawijaya University. Jl. Veteran Malang 65145, East Java, Indonesia; c Essential Oil's Institute, Brawijaya University, Jl Veteran Malang 65145, East Java, Indonesia; d Biosystem Study Center, Brawijaya University, Jl. Veteran Malang 65145, East Java, Indonesia

**Keywords:** Elicitation, Metabarcoding, Mungbean, Ragi Tape, Soybean

## Abstract

*Ragi Tape* (RT) is commonly used as the microbial starter for various Indonesian traditional food. Consist of diverse microbial populations, RT has excellent agents as elicitors to augment the bioactive compounds in Soybean (SB) and Mungbean (MB). Metabarcoding analysis has shown to identify the microbial population involved in elicitation using RT. Inoculated RT from SB and MB were collected to extract the microbial DNA for the post-elicitation group. In comparison, DNA extraction from powdered RT was conducted as a pre-elicitation group. The total DNA were then sequenced by using the MiSeq Illumina platform with 16S rDNA gene region V3-V4 and 18S rDNA gene region V4 as a biomarker for bacterial and fungal identification, correspondingly. The obtained raw-data sequences were then analyzed using QIIME2 pipeline. According to the number of the acquired sequences, the 18S sequencing yielded more DNA strands than the 16S sequencing. However, the number of assigned OTUs was higher in 16S sequences than 18S sequences. From the perspective of the sample, RT has larger distinctive taxa, which were not identified in other samples. This metagenome data will provide fundamental information on RT employment in the elicitation process and further understanding of elicitation mechanisms using RT as biotic elicitors. The data is available at the BioProject database under the NCBI domain with accession no. PRJNA767401.

## Specifications Table

**Table:** 

Subject	*Biotechnology*
Specific subject area	*Metagenomic profiling*
Type of data	Figures, 16S and 18S rDNA amplicon sequencing data
How the data were acquired	Illumina MiSeq platform, QIIME2 2021.4
Data format	Raw Data and Analyzed
Description of data collection	Metagenomic DNA isolated from RT, RT-inoculated soybean, and RT-inoculated mung bean, paired-end amplicon sequencing from V3-V4 16S rDNA gene and V4 18S rDNA gene using Illumina MiSeq
Data source location	• *Institution: Department of Biology, Brawijaya University* • *City/Town/Region: Malang, East Java 65154* • *Country: Indonesia* • *Latitude and longitude: 7°57′4″S 112°36′53″E*
Data accessibility	*Data were available at NCBI Sequence Read Archive (SRA) database under Accession no. PRJNA767401Direct link:*https://www.ncbi.nlm.nih.gov/sra/PRJNA767401
Related research article	F. E. Hermanto, W. Warsito, M. Rifa'i, N. Widodo, Y. D. Jatmiko, Unveiling Microbial Community Structure in Ragi Tape as Elicitors to Increase Secondary Metabolites Contents in Glycine max and Vigna radiata, Biologia. https://doi.org/10.1007/s11756-021-00917-4.

## Value of the Data

• The complete information on bacterial and fungal taxa in *Ragi Tape* will provide valuable information on the development and quality control of *Ragi Tape* production, along with future development for extensive application purposes.
• The data will interest researchers studying conventional biotechnology employing *Ragi Tape* and elicitation to improve the plant's secondary metabolites production.
• The microbial diversity data from these sequence reads may be used to further understand the roles of bacteria and fungi in modulating secondary metabolites production in plant, particularly in host-elicitors interaction during elicitation.
• Several key taxa such as *Pantoea* sp. *Enterobacter* sp., *Bacillus* sp., *Lactobacillus fermentum*, and *Saccharomycopsis fibuligera* provide a promising candidates for future development of secondary metabolites in larger-scale production.

## 1 Data Description

**Fig. 1:**
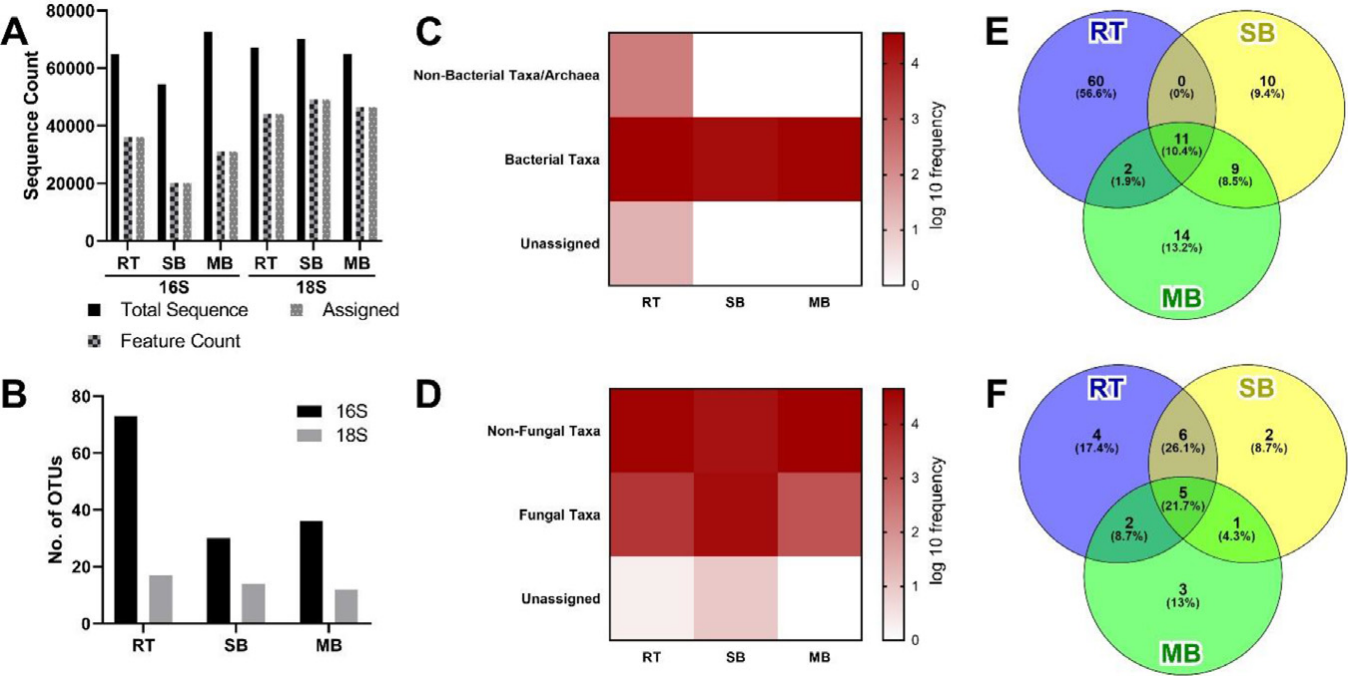
The total number of obtained sequences from sequencing and denoising-filtering step (A) and the number of assigned OTUs from each sample (B). Correspondingly, the frequency of assigned and unassigned sequences in 16S (C) and 18S sequences (D). In addition, the number of distinctive and shared OTUs from 16S (E) and 18S sequences (F) is also described.

The data in this article describe the diversity of bacterial and fungal taxa from RT as elicitors for SB and MB [1]. All of the raw data reads have been deposited on NCBI SRA with the accession number as shown in Table 1. The obtained 16S sequences ranged from 54,519 to 72,571. On the other hand, up to 67,152; 70,104; and 64,938 reads were obtained from the 18S rDNA sequencing from RT, SB, and MB, respectively. Almost half of the 16S raw sequences did not pass the quality control. For 18S sequences, the sequences categorized as candidate taxa (or feature) were 44,126; 49,036; and 46,483 for RT, SB, and MB. However, only 37%-55.63% of 16S sequences and 65.71%-71.58% of 18S sequences were successfully assigned into particular taxa (Fig. 1A). The 16S sequences were assigned greater than 18S sequences, meaning that bacterial populations were more diverse than fungal populations (Fig. 1B). The data also suggest that bacteria have a larger frequency than fungi (Fig. 1C and D). Nevertheless, non-fungal OTUs were also identified from 18S sequences (Fig. 1D). Based on the assigned OTUs in each biomarker, 11 OTUs from 16S sequences were shared in all samples (Fig. 1E). While only 5 OTUs from 18S sequences were co-founded in RT, SB, and MB (Fig. 1F). Interestingly, RT has a higher number of distinctive taxa than other samples, both from 16S or 18S taxonomic assignments (Fig. 1E and F). The detail on the assigned OTUs as described in the table S1 and S2.

**Table 1:** Detail information of every dataset in NCBI SRA database.

No.	Sample Name	Barcode	Treatment	Accession no.
1.	*Ragi Tape*	16S	Control	SRX14239560
2.	*Ragi Tape*	18S	Control	SRX14239561
3.	*Ragi Tape*-elicited soybean	16S	Elicitation	SRX14239562
4.	*Ragi Tape*-elicited soybean	18S	Elicitation	SRX14239563
5.	*Ragi Tape*-elicited mungbean	16S	Elicitation	SRX14239564
6.	*Ragi Tape*-elicited mungbean	18S	Elicitation	SRX14239565

## 2 Experimental Design, Materials and Methods

### 2.1 Samples

*Ragi Tape* (RT) was purchased from a local market in Sawojajar, Malang, East Java, Indonesia. Soybean (SB) var. Anjasmoro and Mungbean (MB) var. Vima-1 were obtained from the Research Center of Legumes and Tuber Plants (BALITKABI), Malang, East Java, Indonesia.

### 2.2 DNA extraction

Elicitation was performed prior to DNA isolation step using the previously described method [1]. Upon elicitation, the beans (SB and MB) were rinsed with sterile distilled water to elute the microbial communities from the beans’ surface. The eluted water was used as DNA isolation samples. The DNA isolation from RT was done by diluting the powdered RT into sterile distilled water (0.1 g/mL) and mixed using vortex for 10 minutes. All samples (SB, MB, and RT) were then passed to the DNA isolation process with DNeasy PowerFood Microbial Kit (Qiagen, Germany) according to manufacturer protocols. The quality of DNA was evaluated using NanoDrop Spectrophotometer and Qubit Fluorometer prior to sequencing step.

### 2.3 Sequencing and raw data processing

Illumina MiSeq (provided by Macrogen, Korea) was employed to sequence the obtained total DNA samples. The bacterial population was identified according to 16S rDNA region V3-V4, while the 18S rDNA region V4 was selected for fungal barcoding. The sequence of primers for 16S and 18S rDNA are as follows: Bakt_341F 5′-CCTACGGGNGGCWGCAG-3′ (forward) and Bakt_805R 5′-GACTACHVGGGTATCTAATCC-3′ (reverse) and V4F 5′- CCAGCAGCCGCGGTAATTCC -3′ (forward) and V4R 5′- ACTTTCGTTCTTGATTAA -3′ (reverse). Raw data sequence then demultiplexed and processed using QIIME2 2021.4 pipeline [2] with DADA2 plugin for quality control handling [3]. Taxonomic assignment was performed by q2-classifier plugin [4] with SILVA version 138 [5] as reference sequence database for bacterial and fungal identification.

## Ethics Statements

This study does not include an animal or human involvement as subjects.

## CRediT Author Statement

**Feri E. Hermanto:** Investigation, Data curation; **Warsito Warsito:** Visualization, Writing – original draft preparation; **Muhaimin Rifa'i:** Writing – review & editing; **Nashi Widodo:** Supervision, Funding acquisition; **Yoga D. Jatmiko:** Methodology, Supervision.

## Declaration of Competing Interest

The authors declare that they have no known competing financial interests or personal relationships that could have influenced the work reported in this paper.

## Data Availability

An Insight of Bacterial and Fungal Population in the Elicitation of Soybean and Mungbean using Ragi Tape (Indonesian Traditional Food Fermenter) (Original data) (NCBI Sequence Read Archive (BioProject)).

## Appendix Supplementary materials

Image, application 1
