# Supplementary material for: Metabarcoding dataset on the elicitation of Soybean and Mungbean using Ragi Tape as elicitors for enhancing secondary metabolites production
Source: Data Brief. 2022 Apr 26;42:108209. doi: 10.1016/j.dib.2022.108209 (PMC9079707; doi:10.1016/j.dib.2022.108209)
Supplement: Supplementary file 1 [file mmc1.pdf]

Table S1. Assigned OTUs from 16S sequences based on the relative frequency of taxa bar plot data

Taxonomy	Relative Frequency		
	RT	SB	MB
d__Bacteria;p__Firmicutes;c__Bacilli;o__Lactobacillales;f__Leuconostocaceae;g__Weissella;__	10801	4	41
d__Bacteria;p__Firmicutes;c__Bacilli;o__Staphylococcales;f__Staphylococcaceae;g__Staphylococcus;__	8460	0	0
d__Bacteria;p__Firmicutes;c__Bacilli;o__Lactobacillales;f__Streptococcaceae;g__Streptococcus;__	6246	0	4
d__Bacteria;p__Firmicutes;c__Bacilli;o__Lactobacillales;f__Lactobacillaceae;g__Lactobacillus;s__Lactobacillus_fermentum	2729	55	149
d__Bacteria;p__Cyanobacteria;c__Cyanobacteriia;o__Chloroplast;f__Chloroplast;g__Chloroplast;__	1754	12	455
d__Bacteria;p__Actinobacteriota;c__Actinobacteria;o__Micrococcales;f__Micrococcaceae;g__Rothia;s__Rothia_aerolata	938	0	0
d__Bacteria;p__Proteobacteria;c__Alphaproteobacteria;o__Rickettsiales;f__Mitochondria;g__Mitochondria;s__Oryza_meridionalis	864	0	0
d__Bacteria;p__Firmicutes;c__Bacilli;o__Bacillales;f__Bacillaceae;g__Bacillus;__	743	5197	139
d__Bacteria;p__Firmicutes;c__Bacilli;o__Lactobacillales;f__Lactobacillaceae;g__Pediococcus;__	655	8	2
d__Bacteria;p__Actinobacteriota;c__Actinobacteria;o__Micrococcales;f__Micrococcaceae;g__Kocuria;s__Rothia_kristinae	410	0	0
d__Bacteria;p__Firmicutes;c__Bacilli;o__Lactobacillales;f__Leuconostocaceae;g__Leuconostoc;__	343	0	0
d__Archaea;__;__;__;__;__;__	209	0	0
d__Bacteria;p__Firmicutes;c__Bacilli;o__Lactobacillales;f__Enterococcaceae;g__Enterococcus;__	199	61	34
d__Bacteria;p__Firmicutes;c__Bacilli;o__Lactobacillales;f__Streptococcaceae;g__Streptococcus;s__Streptococcus_urinalis	193	0	0
d__Bacteria;p__Actinobacteriota;c__Actinobacteria;o__Micrococcales;f__Micrococcaceae;g__Kocuria;__	129	0	0
d__Bacteria;p__Actinobacteriota;c__Actinobacteria;o__Micrococcales;f__Micrococcaceae;g__Rothia;s__Rothia_sp.	118	0	0
d__Bacteria;p__Firmicutes;c__Bacilli;o__Lactobacillales;f__Leuconostocaceae;g__Leuconostoc;s__uncultured_bacterium	102	0	0
d__Bacteria;p__Proteobacteria;c__Gammaproteobacteria;o__Enterobacterales;f__Erwiniaceae;g__Pantoea;__	96	5	11929
d__Bacteria;p__Proteobacteria;c__Gammaproteobacteria;o__Enterobacterales;f__Enterobacteriaceae;g__Enterobacter;__	83	6760	8496
d__Bacteria;p__Cyanobacteria;c__Cyanobacteriia;o__Chloroplast;f__Chloroplast;g__Chloroplast;s__Aegypius_monachus	75	0	0
d__Bacteria;p__Firmicutes;c__Bacilli;o__Lactobacillales;f__Lactobacillaceae;g__Lactobacillus;__	62	0	0
d__Bacteria;p__Cyanobacteria;c__Cyanobacteriia;o__Chloroplast;f__Chloroplast;g__Chloroplast;s__Oryza_sativa	60	0	0
d__Bacteria;p__Firmicutes;c__Bacilli;o__Lactobacillales;f__Streptococcaceae;g__Streptococcus;s__Streptococcus_ferus	56	0	0
d__Bacteria;p__Proteobacteria;c__Alphaproteobacteria;o__Rickettsiales;f__Mitochondria;g__Mitochondria;__	51	0	33
d__Bacteria;p__Proteobacteria;c__Gammaproteobacteria;o__Enterobacterales;f__Enterobacteriaceae;__;__	45	4047	4175
d__Bacteria;p__Firmicutes;c__Bacilli;o__Lactobacillales;f__Streptococcaceae;g__Lactococcus;__	40	0	0
d__Bacteria;p__Firmicutes;c__Bacilli;o__Staphylococcales;f__Staphylococcaceae;g__Macrococcus;__	39	0	0
d__Bacteria;p__Proteobacteria;c__Gammaproteobacteria;o__Enterobacterales;f__Enterobacteriaceae;g__Klebsiella;__	39	0	0
d__Bacteria;p__Bacteroidota;c__Bacteroidia;o__Flavobacteriales;f__Weeksellaceae;g__Chryseobacterium;__	28	0	0
d__Bacteria;p__Firmicutes;c__Bacilli;o__Lactobacillales;f__Streptococcaceae;g__Streptococcus;s__Streptococcus_uberis	26	0	0
d__Bacteria;p__Actinobacteriota;c__Actinobacteria;o__Micrococcales;f__Dermacoccaceae;__;__	23	0	0

Unassigned;__;__;__;__;__;__	23	0	0
d__Bacteria;__;__;__;__;__;__	22	0	0
d__Bacteria;p__Patescibacteria;c__Parcubacteria;o__Parcubacteria;f__Parcubacteria;g__Parcubacteria;s__uncultured_bacterium	21	0	0
d__Bacteria;p__Firmicutes;c__Bacilli;o__Alicyclobacillales;f__Alicyclobacillaceae;g__Alicyclobacillus;__	20	0	0
d__Bacteria;p__Proteobacteria;c__Alphaproteobacteria;o__Acetobacterales;f__Acetobacteraceae;g__Acetobacter;__	19	0	0
d__Bacteria;p__Proteobacteria;c__Gammaproteobacteria;o__Enterobacterales;__;__;__	17	13	68
d__Bacteria;p__Proteobacteria;c__Gammaproteobacteria;o__Burkholderiales;f__Neisseriaceae;g__uncultured;s__uncultured_bacterium	17	0	0
d__Bacteria;p__Actinobacteriota;c__Actinobacteria;o__Micrococcales;f__Dermabacteraceae;g__Brachybacterium;__	17	0	0
d__Bacteria;p__Firmicutes;c__Bacilli;o__Exiguobacterales;f__Exiguobacteraceae;g__Exiguobacterium;__	15	0	0
d__Bacteria;p__Proteobacteria;c__Gammaproteobacteria;o__Enterobacterales;f__Enterobacteriaceae;g__Escherichia-Shigella;__	14	0	0
d__Bacteria;p__Proteobacteria;c__Gammaproteobacteria;o__Aeromonadales;f__Aeromonadaceae;g__Aeromonas;__	14	0	0
d__Bacteria;p__Bacteroidota;c__Bacteroidia;o__Bacteroidales;f__F082;g__F082;__	13	0	0
d__Bacteria;p__Chloroflexi;c__JG30-KF-CM66;o__JG30-KF-CM66;f__JG30-KF-CM66;g__JG30-KF-CM66;s__metagenome	12	0	0
d__Bacteria;p__Proteobacteria;c__Alphaproteobacteria;o__Elsterales;f__uncultured;g__uncultured;__	11	0	0
d__Bacteria;p__Fusobacteriota;c__Fusobacteriia;o__Fusobacteriales;f__Fusobacteriaceae;g__Cetobacterium;__	11	0	0
d__Bacteria;p__Actinobacteriota;c__Actinobacteria;o__Corynebacteriales;f__Corynebacteriaceae;g__Corynebacterium;__	10	0	0
d__Bacteria;p__Proteobacteria;c__Gammaproteobacteria;o__Pseudomonadales;f__Moraxellaceae;g__Acinetobacter;s__Acinetobacter_baumannii	9	35	8
d__Bacteria;p__Proteobacteria;c__Alphaproteobacteria;o__Rhodobacterales;f__Rhodobacteraceae;g__Paracoccus;__	9	0	0
d__Bacteria;p__Deinococcota;c__Deinococci;o__Thermales;f__Thermaceae;g__Meiothermus;__	9	0	0
d__Bacteria;p__Proteobacteria;c__Gammaproteobacteria;o__Salinisphaerales;f__Solimonadaceae;g__Solimonas;__	9	0	0
d__Bacteria;p__Actinobacteriota;c__Actinobacteria;o__Propionibacteriales;f__Propionibacteriaceae;g__Cutibacterium;__	9	0	0
d__Bacteria;p__Proteobacteria;c__Gammaproteobacteria;o__Xanthomonadales;f__Xanthomonadaceae;__;__	9	0	0
d__Bacteria;p__Proteobacteria;c__Gammaproteobacteria;o__Burkholderiales;f__Alcaligenaceae;g__Bordetella;__	7	0	0
d__Bacteria;p__Proteobacteria;c__Gammaproteobacteria;o__Xanthomonadales;f__Rhodanobacteraceae;g__Chujaibacter;s__uncultured_bacterium	7	0	0
d__Bacteria;p__Proteobacteria;c__Alphaproteobacteria;o__Rhizobiales;f__Xanthobacteraceae;g__uncultured;__	7	0	0
d__Bacteria;p__Bacteroidota;c__Bacteroidia;o__Flavobacteriales;f__Flavobacteriaceae;g__Flavobacterium;__	7	0	0
d__Bacteria;p__Firmicutes;c__Clostridia;o__Peptostreptococcales-Tissierellales;f__Peptostreptococcaceae;g__Clostridioides;__	7	0	0
d__Bacteria;p__Patescibacteria;c__Saccharimonadia;o__Saccharimonadales;f__Saccharimonadales;g__Saccharimonadales;__	7	0	0
d__Bacteria;p__Proteobacteria;c__Gammaproteobacteria;o__Xanthomonadales;f__Rhodanobacteraceae;g__Rhodanobacter;__	6	0	0
d__Bacteria;p__Proteobacteria;c__Alphaproteobacteria;o__Rhizobiales;f__Rhizobiaceae;g__Allorhizobium-Neorhizobium-Pararhizobium-Rhizobium;__	6	0	0
d__Bacteria;p__Bacteroidota;c__Bacteroidia;o__Flavobacteriales;f__Weeksellaceae;g__Chryseobacterium;s__Chryseobacterium_hispanicum	6	0	0
d__Bacteria;p__Actinobacteriota;c__Actinobacteria;o__Pseudonocardiales;f__Pseudonocardiaceae;g__Prauserella;s__Prauserella_rugosa	6	0	0
d__Bacteria;p__Actinobacteriota;c__Actinobacteria;o__Micrococcales;f__Brevibacteriaceae;g__Brevibacterium;__	5	0	0
d__Bacteria;p__Bacteroidota;c__Bacteroidia;o__Flavobacteriales;f__Flavobacteriaceae;g__Flavobacterium;s__Flavobacterium_lindanitolerans	5	0	0

d__Bacteria;p__Proteobacteria;__;__;__;__;__	5	0	0
d__Bacteria;p__Proteobacteria;c__Gammaproteobacteria;o__Burkholderiales;f__Comamonadaceae;__;__	5	0	0
d__Bacteria;p__Actinobacteriota;c__Actinobacteria;o__Propionibacteriales;f__Nocardioidaceae;g__Nocardioides;__	4	0	0
d__Bacteria;p__Bacteroidota;c__Bacteroidia;o__Flavobacteriales;f__Weeksellaceae;g__Cloacibacterium;__	4	0	0
d__Bacteria;p__Proteobacteria;c__Gammaproteobacteria;o__Pseudomonadales;f__Moraxellaceae;g__Enhydrobacter;__	4	0	0
d__Bacteria;p__Proteobacteria;c__Alphaproteobacteria;o__Rhodobacterales;f__Rhodobacteraceae;g__Rubellimicrobium;s__Rubellimicrobium_thermophilum	4	0	0
d__Bacteria;p__Proteobacteria;c__Gammaproteobacteria;o__Burkholderiales;f__Methylophilaceae;g__Methylobacillus;__	3	0	0
d__Bacteria;p__Proteobacteria;c__Alphaproteobacteria;o__Sphingomonadales;f__Sphingomonadaceae;g__Sphingomonas;__	3	0	0
d__Bacteria;p__Firmicutes;c__Bacilli;o__Brevibacillales;f__Brevibacillaceae;g__Brevibacillus;__	0	2294	336
d__Bacteria;p__Firmicutes;c__Bacilli;o__Paenibacillales;f__Paenibacillaceae;g__Paenibacillus;__	0	526	169
d__Bacteria;p__Firmicutes;c__Clostridia;o__Clostridiales;f__Clostridiaceae;g__Clostridium_sensu_stricto_1;__	0	358	60
d__Bacteria;p__Proteobacteria;c__Gammaproteobacteria;o__Pseudomonadales;f__Moraxellaceae;g__Acinetobacter;__	0	106	0
d__Bacteria;p__Firmicutes;c__Clostridia;o__Clostridiales;f__Clostridiaceae;g__Clostridium_sensu_stricto_3;__	0	105	0
d__Bacteria;p__Proteobacteria;c__Gammaproteobacteria;o__Pseudomonadales;f__Moraxellaceae;g__Acinetobacter;s__Acinetobacter_schindleri	0	87	0
d__Bacteria;p__Firmicutes;c__Clostridia;o__Clostridiales;f__Clostridiaceae;g__Clostridium_sensu_stricto_5;__	0	83	0
d__Bacteria;p__Firmicutes;c__Bacilli;o__Paenibacillales;f__Paenibacillaceae;__;__	0	82	0
d__Bacteria;p__Proteobacteria;c__Gammaproteobacteria;o__Pseudomonadales;f__Pseudomonadaceae;g__Pseudomonas;__	0	70	14
d__Bacteria;p__Firmicutes;c__Clostridia;o__Lachnospirales;f__Lachnospiraceae;g__Lachnoclostridium;__	0	64	0
d__Bacteria;p__Proteobacteria;c__Alphaproteobacteria;o__Azospirillales;f__Azospirillaceae;g__Azospirillum;__	0	46	55
d__Bacteria;p__Firmicutes;c__Bacilli;o__Bacillales;f__Bacillaceae;g__Bacillus;s__Bacillus_circulans	0	36	263
d__Bacteria;p__Proteobacteria;c__Gammaproteobacteria;o__Burkholderiales;f__Burkholderiaceae;g__Cupriavidus;__	0	35	30
d__Bacteria;p__Firmicutes;c__Bacilli;o__Paenibacillales;f__Paenibacillaceae;g__Paenibacillus;s__Paenibacillus_turicensis	0	27	0
d__Bacteria;p__Firmicutes;c__Bacilli;o__Bacillales;f__Planococcaceae;g__Rummeliibacillus;__	0	26	0
d__Bacteria;p__Firmicutes;c__Bacilli;o__Bacillales;f__Planococcaceae;g__Lysinibacillus;__	0	19	0
d__Bacteria;p__Proteobacteria;c__Gammaproteobacteria;o__Pseudomonadales;f__Moraxellaceae;g__Acinetobacter;s__Acinetobacter_radioresistens	0	6	3391
d__Bacteria;p__Proteobacteria;c__Alphaproteobacteria;o__Rhizobiales;f__Beijerinckiaceae;g__Microvirga;__	0	6	29
d__Bacteria;p__Firmicutes;c__Clostridia;o__Clostridiales;f__Clostridiaceae;__;__	0	6	0
d__Bacteria;p__Proteobacteria;c__Gammaproteobacteria;o__Burkholderiales;f__Oxalobacteraceae;g__Massilia;__	0	0	326
d__Bacteria;p__Proteobacteria;c__Gammaproteobacteria;o__Enterobacterales;f__Enterobacteriaceae;g__Cronobacter;__	0	0	176
d__Bacteria;p__Proteobacteria;c__Gammaproteobacteria;o__Enterobacterales;f__Enterobacteriaceae;g__Kosakonia;__	0	0	125
d__Bacteria;p__Proteobacteria;c__Gammaproteobacteria;o__Enterobacterales;f__Enterobacteriaceae;g__Pseudocitrobacter;__	0	0	115
d__Bacteria;p__Proteobacteria;c__Gammaproteobacteria;o__Xanthomonadales;f__Xanthomonadaceae;g__Xylella;s__Pseudomonas_sp.	0	0	112
d__Bacteria;p__Proteobacteria;c__Gammaproteobacteria;o__Enterobacterales;f__Erwiniaceae;__;__	0	0	95
d__Bacteria;p__Proteobacteria;c__Gammaproteobacteria;o__Xanthomonadales;f__Rhodanobacteraceae;g__Luteibacter;__	0	0	69

d__Bacteria;p__Proteobacteria;c__Gammaproteobacteria;o__Enterobacterales;f__Enterobacteriaceae;g__Franconibacter;s__Franconibacter_helveticus	0	0	61
d__Bacteria;p__Firmicutes;c__Bacilli;o__Paenibacillales;f__Paenibacillaceae;g__Paenibacillus;s__Paenibacillus_relictisesami	0	0	54
d__Bacteria;p__Proteobacteria;c__Gammaproteobacteria;o__Burkholderiales;f__Burkholderiaceae;g__Cupriavidus;s__Cupriavidus_respiraculi	0	0	25
d__Bacteria;p__Proteobacteria;c__Gammaproteobacteria;o__Burkholderiales;f__Burkholderiaceae;g__Burkholderia-Caballeronia-Paraburkholderia;__	0	0	10
d__Bacteria;p__Proteobacteria;c__Alphaproteobacteria;o__Caulobacterales;f__Caulobacteraceae;g__Brevundimonas;__	0	0	7
d__Bacteria;p__Proteobacteria;c__Gammaproteobacteria;o__Xanthomonadales;f__Xanthomonadaceae;g__Xylella;__	0	0	3
d__Bacteria;p__Myxococcota;c__Polyangia;o__Polyangiales;f__Sandaracinaceae;g__uncultured;s__uncultured_bacterium	0	0	2

Table S2. Assigned OTUs from 18S sequences based on the relative frequency of taxa bar plot data

Taxonomy	Relative Frequency		
	RT	SB	MB
d__Eukaryota;p__Phragmoplastophyta;c__Embryophyta;o__Magnoliophyta;f__Magnoliophyta;g__Magnoliophyta;__	38158	18207	45033
d__Eukaryota;p__Ascomycota;c__Saccharomycetes;o__Saccharomycetales;f__Saccharomycopsidaceae;g__Saccharomycopsis;s__Saccharomycopsis_fibuligera	848	22533	983
d__Eukaryota;p__Cercozoa;c__uncultured;o__uncultured;f__uncultured;g__uncultured;s__uncultured_eukaryote	0	2994	0
d__Eukaryota;p__Ascomycota;c__Saccharomycetes;o__Saccharomycetales;f__Debaryomycetaceae;g__Hypopichia-Candida_clade;s__Hyphopichia_burtonii	2550	0	19
d__Eukaryota;p__Ascomycota;c__Eurotiomycetes;o__Eurotiales;f__Aspergillaceae;__;__	80	2016	133
d__Eukaryota;p__Phragmoplastophyta;c__Embryophyta;o__Magnoliophyta;f__Magnoliophyta;g__Magnoliophyta;s__Allium_sativum	1202	0	26
d__Eukaryota;p__Ascomycota;c__Saccharomycetes;o__Saccharomycetales;f__Metschnikowiaceae;g__Clavispora-Candida_clade;s__Clavispora_lusitaniae	0	1097	6
d__Eukaryota;p__Ascomycota;c__Saccharomycetes;o__Saccharomycetales;f__Phaffomycetaceae;g__Cyberlindnera-Candida_clade;__	21	807	7
d__Eukaryota;p__Ascomycota;c__Saccharomycetes;o__Saccharomycetales;f__Metschnikowiaceae;g__Kodamaea;__	617	25	14
d__Eukaryota;p__Ascomycota;c__Saccharomycetes;o__Saccharomycetales;f__Saccharomycetaceae;g__Nakaseomyces-Candida_clade;s__[Candida]_glabrata	33	499	0
d__Eukaryota;p__Ascomycota;c__Saccharomycetes;o__Saccharomycetales;f__Debaryomycetaceae;g__Meyerozyma-Candida_clade;__	78	389	0
d__Eukaryota;p__Ascomycota;c__Saccharomycetes;o__Saccharomycetales;f__Saccharomycetaceae;g__Torulaspora;__	0	278	0
d__Eukaryota;p__Ascomycota;c__Saccharomycetes;o__Saccharomycetales;f__Debaryomycetaceae;g__Millerozyma;s__Millerozyma_farinosa	248	22	0
d__Eukaryota;p__Phragmoplastophyta;c__Embryophyta;o__Magnoliophyta;f__Magnoliophyta;g__Magnoliophyta;s__Phaseolus_acutifolius	0	0	218
d__Eukaryota;p__Ascomycota;c__Saccharomycetes;o__Saccharomycetales;f__Trichomonascaceae;g__Blastobotrys;s__Blastobotrys_adeninivorans	133	0	0
d__Eukaryota;p__Basidiomycota;c__Tremellomycetes;o__Trichosporonales;f__Trichosporonaceae;g__Trichosporon;__	12	108	0
d__Eukaryota;p__Ascomycota;c__Saccharomycetes;o__Saccharomycetales;f__Debaryomycetaceae;g__Candida-Lodderomyces_clade;__	79	0	0
d__Eukaryota;p__Ascomycota;c__Saccharomycetes;o__Saccharomycetales;f__Phaffomycetaceae;g__Wickerhamomyces-Candida_clade;__	32	20	0
d__Eukaryota;p__Vertebrata;c__Mammalia;o__Mammalia;f__Mammalia;g__Mammalia;__	0	0	42
d__Eukaryota;p__Ascomycota;c__Saccharomycetes;o__Saccharomycetales;f__Pichiaceae;g__Pichia;__	10	31	0
d__Eukaryota;p__Ascomycota;c__Saccharomycetes;o__Saccharomycetales;f__Saccharomycopsidaceae;g__Saccharomycopsis;s__Saccharomycopsis_malanga	13	0	0
d__Eukaryota;p__Mucoromycota;c__Incertae_Sedis;o__Mucorales;f__Rhizopodaceae;g__Rhizopus;__	10	0	0
Unassigned;__;__;__;__;__;__	2	10	0

d__Eukaryota;p__Arthropoda;c__Maxillopoda;o__Calanoida;f__Calanoida;g__Calanoida;__	0	0	2
